# Transcriptional Activation of Estrogen Receptor-Alpha and Estrogen Receptor-Beta from Elephant Shark (*Callorhynchus milii*)

**DOI:** 10.3390/genes17030327

**Published:** 2026-03-17

**Authors:** Ya Ao, Haruka Narita, Wataru Takagi, Susumu Hyodo, Michael E. Baker, Yoshinao Katsu

**Affiliations:** 1 Graduate School of Life Science, Hokkaido University, Sapporo 060-0810, Japan; aoya19960720@gmail.com (Y.A.); hrkn47@gmail.com (H.N.); 2 Laboratory of Physiology, Atmosphere and Ocean Research Institute, The University of Tokyo, Chiba 277-8564, Japan; 3 Division of Nephrology-Hypertension, Department of Medicine, University of California San Diego, 9500 Gilman Drive, La Jolla, CA 92093-0693, USA; 4 Center for Academic Research and Training in Anthropogeny (CARTA), University of California San Diego, La Jolla, CA 92093-0693, USA; 5 Faculty of Science, Hokkaido University, Sapporo 060-0810, Japan

**Keywords:** elephant shark, estrogen receptor evolution, ERa, ERb

## Abstract

Background/Objectives: Humans and other vertebrates contain two estrogen receptors (ERs), ERa and ERb. Among cartilaginous fish (sharks, rays, skates), which are chondrichthyans that evolved about 425 million years ago, only activation by steroids of ERb orthologs has been characterized. To remedy this gap in understanding estrogen signaling in chondrichthyans, we studied estrogen activation of orthologs of human ERa and ERb from elephant shark (*Callorhynchus milii*). Methods/Results: Unexpectedly, we found that *C. milii* contained three estrogen-responsive ERa genes: ERa1 (596 amino acids), ERa2 (600 amino acids), and ERa3 (599 amino acids) with strong sequence similarity to each other. We also found an estrogen-unresponsive gene, ERa4 (561 amino acids), with a 39 amino acid deletion in the DNA-binding domain. An estrogen-responsive ERb ortholog (580 amino acids) was also present in *C. milii*. The three active *C. milii* ERas are of similar length to human ERa (595 amino acids); however, *C. milii* ERb is longer than human ERb (530 amino acids). We studied transcriptional activation of ERa and ERb by estradiol (E2), the main reproductive estrogen in humans. We also studied estrone (E1), the main postmenopausal estrogen, and estriol (E3), which is synthesized during pregnancy. We determined the half-maximal response (EC50) and fold-activation to E2, E1, and E3 of *C. milii* ERa1, ERa2, ERa3, and ERb. Among these estrogens, E2 had the lowest EC50 for all four ERs. Fold-activation by E2 and E3 was similar for ERa1, ERa2, ERa3, and ERb. Conclusions: Overall, estrogen activation of *C. milii* ERa and ERb was similar to that for human ERa and ERb, indicating substantial conservation of the vertebrate ER during the 425 million years since the divergence of cartilaginous fish and humans from a common ancestor.

## 1. Introduction

Estrogens are a class of steroid hormones that have diverse physiological activities in females and males in humans and other vertebrates [1,2,3,4,5]. Estrogens act by binding to estrogen receptors (ERs) [5,6], which are transcription factors that belong to the nuclear receptor family, which also includes receptors for progestins, androgens, and corticosteroids [7,8,9]. To date, two distinct estrogen receptor genes, estrogen receptor alpha (ERa) and estrogen receptor beta (ERb), have been isolated in mammals and other vertebrates [10,11,12]. ERα and ERb have strong sequence similarity (97%) in their DNA-binding domains, but only about 55% similarity in their ligand-binding domains.

**Figure 1:**
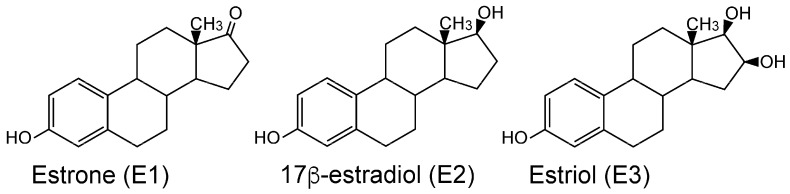
**Structures of estrogen.** Estrone (E1), 17b-estradiol (E2), and estriol (E3) are the natural estrogens. E1 is a minor female sex hormone and serves mainly as a precursor of E2. E3 is produced in the placenta during pregnancy. E2 is a major female sex hormone mainly produced in the ovary [14,15].

ERα is expressed in reproductive tissues (uterus, ovary), breast, kidney, and bone, while ERβ is expressed in the ovary and male reproductive organs (prostate), colon, kidney, and the immune system. The response of ERa and ERb in humans and other vertebrates to 17b-estradiol (E2), the main physiological estrogen, as well as to estrone (E1), a postmenopausal estrogen, and estriol (E3), which is synthesized during pregnancy (Figure 1), has been studied extensively [5,6,10,13]. Although ERα and ERβ can have opposing actions, both receptors are activated by binding estrogens, which induce a conformational change that promotes binding to specific DNA sequences and the initiation of estrogen-dependent gene transcription. ERα promotes tissue proliferation, while ERβ may act as a suppressor of ERα-mediated proliferation.

Although ERs with sequence similarity to ERa [16,17] and ERb [16,17,18] have been isolated from elasmobranchs (sharks, rays, and skates), only the response to steroids by ERb from the cloudy catshark (*Scyliorhinus torazame*) and whale shark (*Rhincodon typus*) has been studied [18]. Estrogen activation of a gnathostome ERa has not been reported. Elasmobranchs, along with Holocephali, comprise the two extant subclasses of Chondrichthyes, jawed fishes with skeletons composed of cartilage rather than calcified bone [19]. Chondrichthyes occupy a key position relative to fish and terrestrial vertebrates, having diverged from bony vertebrates about 450 million years ago [16,20]. As the oldest group of extant jawed vertebrates (gnathostomes), the properties of ERa and ERb in cartilaginous fishes can provide important insights into the early evolution of ERa and ERb from an ancestral ER in lamprey [21]. Moreover, elephant shark has the slowest evolving genome of all known vertebrates, including the slowly evolving coelacanth [16], which makes elephant shark genes a good system to study the evolution of the ER, including the early evolution of selectivity of the ER in a vertebrate and the subsequent divergence in steroid selectivity in human ER, in other vertebrate ERs and in ray-finned fish ERs [22]. Another point of interest for the ER in elephant shark is that elephant sharks contain three full-length ERas [16], raising the question of how these three ERas compare with each other and with mammalian ERas in their response to estrogens.

To remedy this gap in our understanding of ERa activation in vertebrates, we have studied the response to estradiol, estrone, and estriol of three full-length *Callorhinchus milii* ERas, as well as *C. milii* ERb. Here, we report that despite strong sequence similarity among *C. milii* ERa1, ERa2, ERa3, and ERb, there are some differences in their half-maximal response (EC50) and fold-activation to these three physiological estrogens. We find that E2 had the lowest EC50 for all four ERs. E2 and E3 had a similar fold activation for ERa1, ERa2, ERa3, and ERb. Overall, estrogen activation of *C. milii* ERa and ERb was similar to that for human ERa and ERb [5,7], indicating substantial conservation of the vertebrate ER in the 425 million years since the divergence of cartilaginous fish and humans from a common ancestor.

## 2. Materials and Methods

### 2.1. Animals

*C. milii* elephant sharks of both sexes were collected in Western Port Bay, VIC, Australia, using recreational fishing equipment, and transported to Primary Industries Research Victoria, Queenscliff, using a fish transporter. The animals were kept in a 10 t round tank with running seawater (SW) under a natural photoperiod for at least several days before sampling. Animals were anesthetized in 0.1% (*w*/*v*) 3-amino benzoic acid ethyl ester (Sigma-Aldrich, St. Louis, MO, USA). After the decapitation of the animal, tissues were dissected out, quickly frozen in liquid nitrogen, and kept at −80 °C. All animal experiments were conducted according to the Guideline for Care and Use of Animals approved by the committees of the University of Tokyo, as described in [23] [Animal Ethics Committee of the Ocean Research Institute of the University of Tokyo], approval code: [17-3], and approval date: [29 March 2005].

### 2.2. Chemical Reagents

Estrone (E1), 17b-estradiol (E2), and estriol (E3) were purchased from Sigma-Aldrich Corp. (St. Louis, MO, USA). All chemicals were dissolved in dimethyl sulfoxide (DMSO). The concentration of DMSO in the culture medium did not exceed 0.1%.

### 2.3. Cloning of Estrogen Receptors

When we began this project, the genome of *C. milii* had not yet been sequenced [16]. Thus, we needed to sequence an ER in *C. milii* as a first step in determining the number of ERs and their sequences in *C. milii*. For this goal, we used two conserved amino acid regions in the DNA-binding domain (GYHYGVW) and the ligand-binding domain (NKGM/IEH) of vertebrate ERs to design degenerate oligonucleotides to clone *C. milii* ERs. The second PCR used the first PCR amplicon and nested primers that were selected in the DNA-binding domain (CEGCKAF) and the ligand-binding domain (NKGM/IEH). As a template for PCR, the first-strand cDNA was synthesized using total RNA isolated from the ovary. The amplified DNA fragments were subcloned with TA-cloning plasmids pCR2.1-TOPO (Invitrogen, Carlsbad, CA, USA). The 5′ and 3′ ends of the ER cDNAs were amplified by rapid amplification of the cDNA end (RACE) using a SMART RACE cDNA Amplification kit (BD Biosciences Clontech, Palo Alto, CA, USA). To amplify the isoforms of elephant shark ER, we applied a PCR-based cDNA amplification technique using the primers et (5′-TGAAGTGTGATCGTCCAGGCGACAG-3′ and 5′-CAAGCTGGAGGATAAGACATCGAC-3′). Sequencing was performed using a BigDye Terminator Cycle Sequencing kit and analyzed on the Applied Biosystems 3730 DNA Analyzer (Thermo Fisher Scientific Inc., Waltham, MA, USA)

We used the *C. milii*-extracted RNA to clone ERa1, ERa2, ERa3, ERa4, and ERb, which had sequences corresponding to the sequences of the *C. milii* genome deposited by Venkatesh et al., 2014 [16].

### 2.4. Database and Sequence Analyses

All sequences generated were searched for similarity using BLASTn (https://blast.ncbi.nlm.nih.gov/Blast.cgi (accessed on 8 December 2025) and BLASTp at the web servers of the National Center of Biotechnology Information (NCBI). We found four ERa genes and one ERb gene [16], which were aligned using ClustalW [24].

### 2.5. Phylogenetic Tree Analysis

The evolutionary history was inferred using the neighbor-joining method [25]. The optimal tree is presented in this study. The percentage of replicate trees in which the associated taxa clustered together in the bootstrap test (1000 replicates) is shown next to the branches [26]. The tree is drawn to scale, with branch lengths in the same units as those of the evolutionary distances used to infer the phylogenetic tree. The evolutionary distances were computed using the Poisson correction method and are in units of the number of amino acid substitutions per site. Evolutionary analyses were conducted in MEGA 11 [27].

### 2.6. Reporter Gene Assay

Full-length estrogen receptors were amplified using specific forward and reverse primers designed at start and stop codons and cloned into the mammalian expression vector pcDNA3.1 (Invitrogen). In addition, ERs with a FLAG-tag added to the N-terminus were also prepared by PCR. A reporter construct, pGL4.23-4xERE, was produced by subcloning of oligonucleotides having 4xERE into the *Kpn*I-*Hind*III site of pGL4.23 vector (Promega, Madison, WI, USA). All cloned DNA sequences were verified by sequencing.

Reporter gene assays using full-length ERs were performed in Human Embryonic Kidney 293 cells (HEK293 cells). HEK293 cells were seeded in 24-well plates at 5 × 10^4^ cells/well in phenol-red-free Dulbecco’s modified Eagle’s medium with 10% charcoal/dextran-treated fetal bovine serum. After 24 h, the cells were transfected with 400 ng of reporter construct, 25 ng of pRL-TK (as an internal control to normalize the variation in transfection efficiency; contains the *Renilla reniformis* luciferase gene with the herpes simplex virus thymidine kinase promoter), and 200 ng of pcDNA3.1-estrogen receptor using polyethylenimine (PEI). After 5 h of incubation, ligands were applied to the medium at various concentrations. After an additional 43 h, the cells were collected, and the luciferase activity of the cells was measured with the Dual-Luciferase Reporter Assay System. Promoter activity was calculated as firefly (*Photinus pyralis*) luciferase activity/sea pansy (*R. reniformis*) luciferase activity. The values shown are mean ± SEM from three separate experiments, and dose–response data, which were used to calculate the half-maximal response (EC50) for each steroid, were analyzed using GraphPad Prism (Version 11, GraphPad Software, Inc., San Diego, CA, USA). All experiments were performed in triplicate.

## 3. Results

### 3.1. Cloning and Sequence Analysis of cDNAs for Four Elephant Shark ERa Genes and One ERb Gene

**Figure 2:**
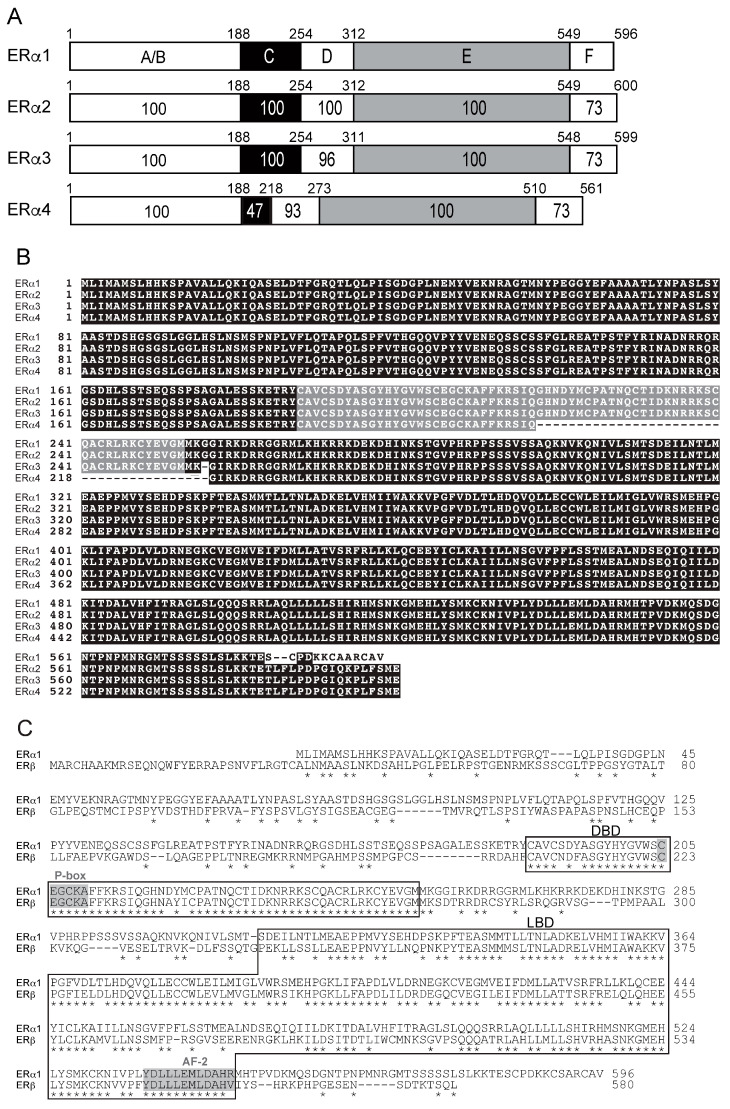
**Amino acid sequences of the elephant shark ERs.** (**A**) Comparison of functional domains of four elephant shark ERa genes. Elephant shark ERa1, ERa2, and ERa3 show strong sequence conservation of their A/B, C, D, and E domains. The function of the F domain is not well understood. ERa4 has a deletion in the DNA-binding domain [C domain], which we propose explains its lack of transcriptional activation by estradiol. (**B**) Sequence alignment of elephant shark ERa1, ERa2, ERa3, and ERa4. There is excellent conservation of the four elephant shark ERa genes. (**C**) Aligned sequences of the elephant shark ERa1 and ERb. The DBD and LBD are indicated by open boxes. Residues are important for DNA response element recognition (the P-box) and the AF-2 region, which mediates contact of the LBD with transcriptional coactivators (shaded in gray). Numbers to the right indicate amino acid position. Asterisks * indicate residues conserved in both ERa1 and ERb.

Using standard RT-PCR and RACE techniques, we successfully cloned four full-length ERs, designated as ERa1, ERa2, ERa3 (Figure 2A,B), and ERb (Figure 2C), from elephant shark ovary RNA. The cDNA for elephant shark ERa1 predicted a 596 amino acid protein with a calculated molecular mass of 66.6 kDa (GenBank accession no. LC068847), and the cDNA for elephant shark ERb predicted a 580 amino acid protein with a calculated molecular mass of 64.9 kDa (GenBank accession no. LC068848), Figure 2A,B. In addition, we cloned the other two full-length elephant shark ERs: ERa2 and ERa3, which are found in GenBank (GenBank accession no. XM_007894403 for ERa2, XM_007894404 for ERa3). ERa4 (GenBank accession no. XM_007894406) with a 39-amino-acid deletion in the DNA-binding domain was also cloned (Figure 2A,B).

The elephant shark ERa and ERb sequences contain the five recognizable steroid hormone receptor sub-domains in the expected order: the N-terminal region (A/B domain), DNA-binding domain, DBD (C domain), hinge region (D domain), ligand-binding domain, LBD (E domain), and C-terminal extension (F domain) (Figure 2A). In Figure 2A, we compare the functional domains of ERa1, ERa2, ERa3, and ERa4. Figure 2A shows that there is excellent conservation among these ERs of the A/B, C, D, E, and F domains. This strong sequence conservation is also seen in the amino acid alignment of ERa1, ERa2, ERa3, and ERa4 shown in Figure 2B. Figure 2B shows the deletion in the DBD of ERa4, which probably explains the lack of transcriptional activation by estrogens of *C. milii* ERa4. The amino acid sequence of ERa4 is closest to ERa3. Overall, Figure 2A,B show the strong sequence conservation in the four *C. milii* ERa genes.

In Figure 2C, we show a sequence alignment of *C. milii* ERa1 and ERb. The DNA response element recognition motif (P-box, CEGCKA) in the DBD and the AF-2 motif (core LLLEML region) in the LBD, which is required for interaction with transcriptional co-activators, are conserved in all four elephant shark ERa sequences and in ERb (Figure 2B,C).

### 3.2. Comparison of Functional Domains on Human ERa and ERb with Functional Domains in Elephant Shark ERa and ERb

**Figure 3:**
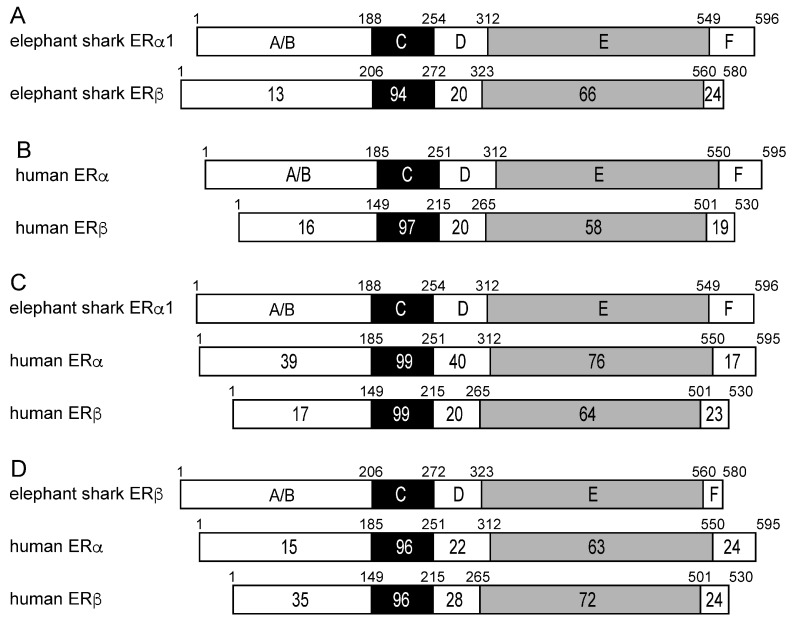
**Comparison of the functional domains of elephant shark ERs with human ERs.** The functional domains A/B through F are shown schematically with the number of amino acid residues indicated. The percentage of amino acid identity is shown. (**A**) Comparison of elephant shark ERa1 with elephant shark ERb. The N-terminal (A/B) domains of elephant shark ERa1 and ERb show strong divergence. There is also some divergence between the ligand binding domain (E)- on elephant shark ERa1 and ERb. (**B**) Comparison of human ERa (accession no. NM000125) with human ERb (accession no. AB006590). The domains of human ERa and human ERb have sequences similar to each other, as elephant shark ERa1 and ERb. (**C**) Comparison of elephant shark ERa1 with human ERa and human ERb. Elephant shark ERa1 is closer to human ERa than to human ERb. (**D**) Comparison of elephant shark ERb with human ERa and human ERb. Elephant shark ERb is closer to human ERb than to human ERa.

To gain additional insights into the evolution of the *C. milii* ERs, we compared the functional domains of elephant shark ERa1 to elephant shark ERb (Figure 3A), of elephant shark ERa1 to human ERa and human ERb (Figure 3C), and of elephant shark ERb to human ERa and human ERb (Figure 3D). We also compared human ERa and ERb to each other (Figure 3B).

Figure 3A shows the excellent conservation of the DNA-binding domains [C domains] (94% identity) and ligand-binding domains [E domains] (66% identity) in elephant shark ERa1 and ERb, in contrast to the weak conservation of the A/B domains (13% identity) at their N-terminus. Comparison of Figure 3A,B reveals that the DBD in human ERa and *C. milii* ERa1 and ERb have similar sequence conservation, while the LBD sequence in *C. milii* ERa1 and *C. milii* ERb is more conserved (66% identity) than the LBD in human ERa and ERb (58% identity).

Figure 3C shows the strong sequence conservation of the DBD in elephant shark ERa1 and human ERa and ERb. Figure 3C also shows that there is stronger conservation (76%) of the LBD in elephant shark ERa1 and human ERa, compared to the similarity (64%) of the corresponding LBD in elephant shark ERa1 to human ERb. Figure 3D shows the strong sequence conservation of the DBD in elephant shark ERa1 and human ERa and ERb. Figure 3D also shows the stronger conservation (72%) of the LBD in elephant shark ERb and human ERb, compared to the similarity (64%) to the corresponding LBD in human ERa.

### 3.3. Comparison of Functional Domains on Elephant Shark ERa and ERb with Functional Domains in Zebrafish, and Whale Shark

**Figure 4:**
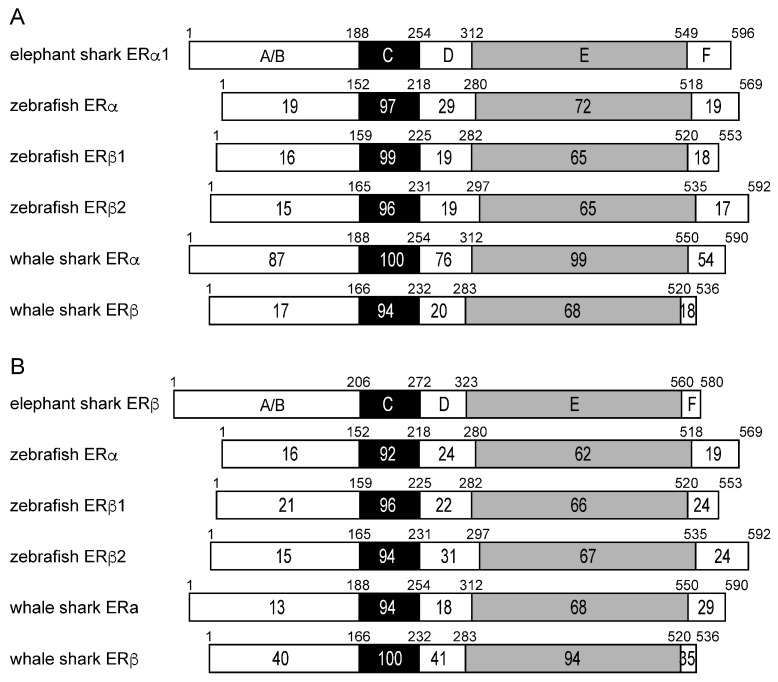
Comparison of the functional domains of elephant shark ERs with ERs from zebrafish and whale shark. The functional domains A/B through F are shown schematically with the number of amino acid residues indicated. The percentage of amino acid identity is shown. (**A**) Comparison of elephant shark ERa1 with zebrafish and whale shark ERs. (**B**) Comparison of elephant shark ERb with zebrafish and whale shark ERs. GenBank accession no. NM_152959 (zebrafish ERa), AF516874 (zebrafish ERb1), AF349413 (zebrafish ERb2), XM_048600891 (whale shark ERa), and AB551716 (whale shark ERb).

To gain an additional insight into the evolution of elephant shark ERa and ERb, we compared their sequences to ERa and ERb sequences in zebrafish and whale shark (Figure 4).

The sequence comparisons (Figure 4) between elephant shark ERa and ERb domains with ERa and ERb domains in whale shark revealed an unexpected sequence similarity (87% identity) between the A/B domain on elephant shark ERa and whale shark ERa. The A/B domain in whale shark ERb and elephant shark ERb has 40% identity. Interestingly, the A/B domains on elephant shark ERa and ERb domains have 39% and 35% sequence identity, respectively, with the A/B domains on human ERa and human ERb, respectively.

The stronger conservation of the LBD in elephant shark ERb and human ERb (72% identity), compared to the similarity to the corresponding LBD in human ERa (63% identity), is shown in Figure 3C. The LBD of elephant shark ERa1 is closer to the LBD in human ERa than the LBD in human ERb, and the LBD of elephant shark ERb is closer to the LBD in human ERb than the LBD in human ERa (Figure 3C).

### 3.4. Phylogenetic Analysis of Estrogen Receptors in Elephant Sharks, Humans, and Other Vertebrates

**Figure 5:**
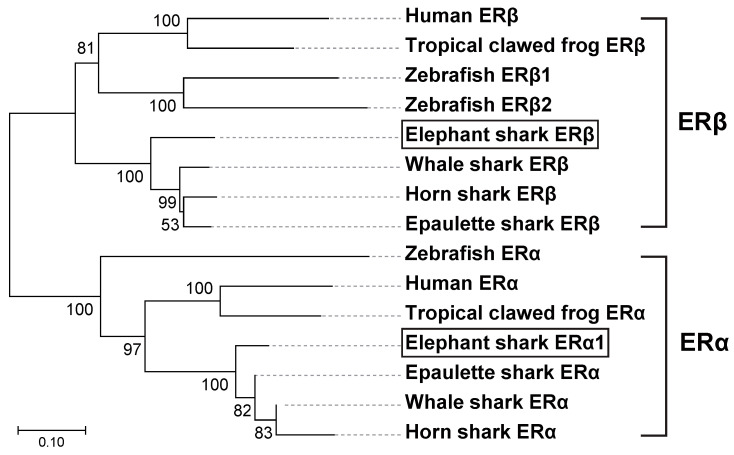
**Phylogenetic analysis of estrogen receptors in elephant sharks, humans, and other vertebrates.** The phylogenetic tree was constructed using the maximum likelihood analysis [26]. The two elephant shark ERs are marked with open boxes. The scale bar indicates 0.1 expected amino acid substitutions per site. The GenBank accession numbers are NM_000125 (human ERa), AB006590 (human ERb), NM_203535 (tropical clawed frog ERa), NM_001040012 (tropical clawed frog ERb), XM_048600891 (whale shark ERa), AB551716 (whale shark ERb), XM_068044449 (horn shark ERa), XM_068038484 (horn shark ERb), XM_060831156 (epaulet shark ERa), XM_060829508 (epaulet shark ERb), NM_152959 (zebrafish ERa), AF516874 (zebrafish ERb1), AF349413 (zebrafish ERb2).

To better understand the relationship between elephant shark ERs and other vertebrates’ ERs, we constructed a phylogeny of ERs from sharks, humans, and other vertebrates (Figure 5) using the neighbor-joining method [25]. The phylogeny shows the early divergence of shark ERs from other ERs.

### 3.5. Transcriptional Activities of Elephant Shark ERa and ERb

**Figure 6:**
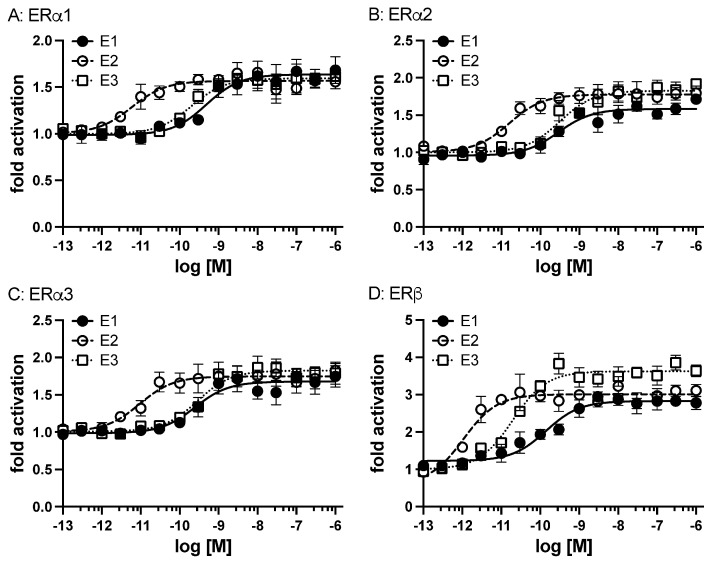
**Transcriptional activation of elephant shark ERs.** Elephant shark ERs were transfected into HEK293 cells with an ERE-driven reporter gene. Concentration–response profile for ERa1 (**A**), ERa2 (**B**), ERa3 (**C**), and ERb (**D**) for E1, E2, and E3 (10^−13^ M to 10^−6^ M). Data are expressed as a ratio of a vehicle (DMSO) to other test chemicals. Each point represents the mean of triplicate determinations, and vertical bars represent the mean ± SEM.

A transactivation assay was used to examine the response to the physiological estrogens E1, E2, and E3 of the three active isoforms of elephant shark ERa. Among the three isoforms, ERa showed ligand-dependent transactivation (Figure 6A–C). The EC50s for transcriptional activation of ERa1, ERa2, and ERa3 were 0.49 nM, 0.29 nM, and 0.25 nM (E1), 0.0059 nM, 0.016 nM, and 0.0093 nM (E2), and 0.2 nM, 0.31 nM, and 0.29 nM (E3), respectively (Table 1).

We also examined the induction of elephant shark ERb transcriptional activity by different concentrations of estrogens (E1, E2, and E3) (Figure 6). All three estrogens activated transcription of elephant shark ERb in a dose-dependent manner (Figure 6D). The half-maximal response (EC50) for transcriptional activation of elephant shark ERb was 0.14 nM for E1, 0.001 nM for E2, and 0.02 nM for E3 (Table 1). Although there was no significant difference in EC50 values for activation by an estrogen of ERa1 and ERb, the fold-activation values for E2 were higher than those for E1 and E2 (Table 1). Thus, the elephant shark estrogen receptors exhibited a ligand responsiveness observed for human estrogen receptors [1,2,3,4,5,6,7,8,9,10].

## 4. Discussion

Significant advances have been made in elucidating the evolution of estrogen signaling in animals [1,2,9,11,21,28,29]. Amphioxus is a chordate that contains an ER and a steroid receptor (SR) that is an ortholog of receptors for 3-ketosteroids, including testosterone, progesterone, cortisol, and aldosterone [11,30]. Contrary to expectations, the amphioxus ER does not bind E2 or other steroids [11,29,30], while E2 and E1 are transcriptional activators of the SR [11,30]. Atlantic sea lamprey contains an ER [21] that is activated by E2 and E1 [11,29].

The estrogen receptors in Chondrichthyes, cartilaginous fishes with jaws, are basal to lobe-finned fishes and are ancestors of terrestrial vertebrates [16]. These ERs are still not fully characterized [17,31]. The identification of ERa1, ERa2, ERa3, ERa4, and ERb from the sequencing of the elephant shark [16] provided an opportunity to investigate estrogen signaling in a basal jawless vertebrate that evolved about 425 million years ago. We investigated the response to E1, E2, and E3 of ERa1, ERa2, ERa3, and ERb from elephant shark expressed in HEK293 cells (Figure 5). These studies revealed that elephant shark ERa1, ERa2, ERa3, and ERb are activated by E1, E2, and E3. We note that we found that ERa4 did not respond to an estrogen), which we propose is due to a sequence deletion in the DBD. Thus, we did not continue studies of estrogen activation of ERa4.

Our data with estrogen activation of ERa1, ERa2, ERa3, and ERb align with the established ligand-binding characteristics of ERs in other vertebrates, thereby substantiating the conserved nature of estrogen signaling pathways [30,32,33,34]. Estrogen-dependent transcriptional activity of the ERs from various species, including other sharks, revealed that the ERs from all species have a stronger response to E2 than to E3 [18,35,36,37,38,39].

Instead of a single ERa gene, elephant shark contains three ERa genes with very similar sequences, which is unexpected. The strong sequence conservation of ERa4, which is not ligand-activated, is also unexpected. Further research is needed to gain a more comprehensive understanding of the physiological function of estrogen and the multiple estrogen receptors in elephant sharks. This data should provide clues for understanding the physiological function(s) of each isoform.

In summary, this is the first report with evidence for the conservation of elephant shark ERa and ERb compared to human ERa and ERb, which provides a valuable perspective on the early evolutionary history of these critical nuclear receptors in vertebrates. The findings from this study not only confirm the conserved nature of ER-mediated estrogen signaling across vertebrates but also open new avenues for research into the functional diversification of these receptors across vertebrates. These findings furnish researchers with critical molecular data to examine the role of ERs in future studies, such as those examining gonadal development, reproductive biology, and developmental and evolutionary endocrinology.

## Figures and Tables

**Table 1:** Gene transcriptional activities of elephant shark ERs by estrogens in HEK293.

		E1	E2	E3
ERa1	EC50 (nM)	0.49	0.0059	0.2
	Fold-Activation (±SEM) *	1.62 (±0.16)	1.66 (±0.04)	1.57 (±0.09)
	Ratio **	0.98	1.00	0.95
ERa2	EC50 (nM)	0.29	0.016	0.31
	Fold-Activation (±SEM) *	1.51 (±0.12)	1.79 (±0.12)	1.82 (±0.13)
	Ratio **	0.84	1.00	1.02
ERa3	EC50 (nM)	0.25	0.0093	0.29
	Fold-Activation (±SEM) *	1.55 (±0.11)	1.75 (±0.08)	1.87 (±0.15)
	Ratio **	0.89	1.00	1.07
ERb	EC50 (nM)	0.14	0.001	0.02
	Fold-Activation (±SEM) *	2.87 (±0.16)	3.23 (±0.26)	3.49 (±0.26)
	Ratio **	0.89	1.00	1.08

* Fold-activation values were calculated based on the 10 nM values. ** Ratio values were calculated by dividing the fold-activation value of each estrogen by the value of E2.

## Data Availability

All data are available on request to Dr. Katsu or to Dr. Baker.
